# Therapeutic success in fragmented coronoid process disease and other canine medial elbow compartment pathology: a systematic review with meta-analyses

**DOI:** 10.3389/fvets.2023.1228497

**Published:** 2023-11-09

**Authors:** Hubertus Kähn, Yury Zablotski, Andrea Meyer-Lindenberg

**Affiliations:** ^1^Clinic of Small Animal Surgery and Reproduction, Centre for Clinical Veterinary Medicine, LMU Munich, Munich, Germany; ^2^Centre for Clinical Veterinary Medicine, LMU Munich, Munich, Germany

**Keywords:** dog, elbow dysplasia, systematic review, meta-analysis, arthroscopy, medial coronoid process, medial compartment disease

## Abstract

**Introduction:**

The correct treatment of elbow dysplasia is controversial in modern small animal orthopedics. The aim of this study was to compile all relevant literature of the therapy of fragmented coronoid process and other hereditary disorders of the medial elbow compartment and to statistically evaluate the therapeutic results in three meta-analyses.

**Methods:**

The basis for the systematic literature review was a comprehensive database search of Web of Science, PubMed and Medline. Studies on living patients with above mentioned degenerative joint disease were included in the initial literature search. The data from the final studies, selected according to the PRISMA guidelines, was subsequently extracted. Finally, the success of the different therapies was compared and analyzed by three meta-analyses: success rate, mean difference and standardized mean difference.

**Results:**

Fourteen of 494 publications covered by the systematic literature search remained. Their overall truth was: In studies where surgery outcomes was determined by clinical examination and owner questionnaires, it was found that surgical intervention had a significant positive outcome in the presence of fragmented coronoid process and medial compartment disease. Surgical outcomes were also good in three cross-over studies that investigated treatment success using computerized gait analysis. In contrast, comparative studies between surgical and conservative management yielded controversial results. The meta-analysis found no significant difference between medical and surgical therapy.

**Discussion:**

The positive results of studies investigating owner satisfaction and veterinary clinical examination of surgical therapy for medial compartment disease were confirmed by two meta-analyses. However, their study designs were susceptible to observer biases. A third meta-analysis of standardized mean difference differentiating computerized gait analysis results of surgical and conservative management found no evidence of significant superiority of each treatment modality. It however had a limited number of subjects. More comparative studies of high evidence are needed to better understand medial compartment disease and provide the clinician with more accurate diagnostics to separate pathology that should be treated surgically from pathology that can benefit from conservative therapy similarly. Given the invasiveness a more cautious approach might be warranted regarding generally recommending surgery for pathology of the medial elbow compartment.

## 1. Introduction

Elbow dysplasia (ED), a hereditary elbow joint abnormality occurs frequently in growing dogs of large and very large breeds. In many cases, both limbs are affected. Male dogs are affected about twice as often as bitches (1). It is a disease complex that includes four pathologies according to the IEWG (International Elbow Working Group) (2). These are fragmented coronoid process, osteochondrosis of the medial humeral condyle, ununited anconeal process, and elbow joint incongruity. The fragmented coronoid process accounts for 65% of all cases of elbow dysplasia, making it the most common type of lesion causing ED (3). However, other pathologies of the medial elbow compartment can also cause similar pain and lameness. Recent research and studies investigating arthroscopy findings of the medial elbow included cartilage fibrillation and erosions, chondromalacia-like lesions, non-displaced fragments and fissures among others (4, 5). Despite that they are mostly a byproduct of the common fragmented coronoid process they can also exist solely. Rigorous differentiation with high quality imaging modalities preferably arthroscopy has been lacking in even quite recent publications. Therefore, present study includes all pathologic findings in the medial elbow compartment from now on termed medial compartment disease (MCD). Persistent intermittent forelimb lameness is typically the initial symptom observed in most cases of MCD in dogs between 6 and 12 months of age. Less commonly, dogs are affected later in life (over 6 years of age). These patients may only show clinical signs at that point and occasionally may not have any prior history of lameness (4, 5). There is disagreement as to which surgical method is superior or if conservative treatment is a viable alternative. This systematic review focuses on evaluating the effectiveness of surgical treatment for the medial elbow compartment in dogs and lastly aims to compare it to less invasive medical management.

## 2. Methods

### 2.1. Database search

The following databases were selected for the literature search: Web of Science Core Collection, PubMed and Medline. MeSH-terms and keywords were picked and various tools such as Boolean operators and truncation were used. This was done to make the search as specific as possible. In addition, the online dissertation and habilitation index of the German National Library was searched with various synonyms and partial synonyms of “dog” and “fragmented coronoid process” or “medial compartment disease” as well as manually the bibliographies of other relevant publications on the topic. This review covers research up to August 2022, the date of the last database search. It was designed so that all papers covered by the search contained the following three generic terms including words related in meaning: Dog, Elbow Disease, Therapy. Individual search strategies were devised for PubMed, Web of Science, and Medline and are listed in Supplementary data S1–S3. Subsequently, all duplicates that were present simultaneously in two or more databases were removed.

### 2.2. Identification of relevant literature

**Figure 1:**
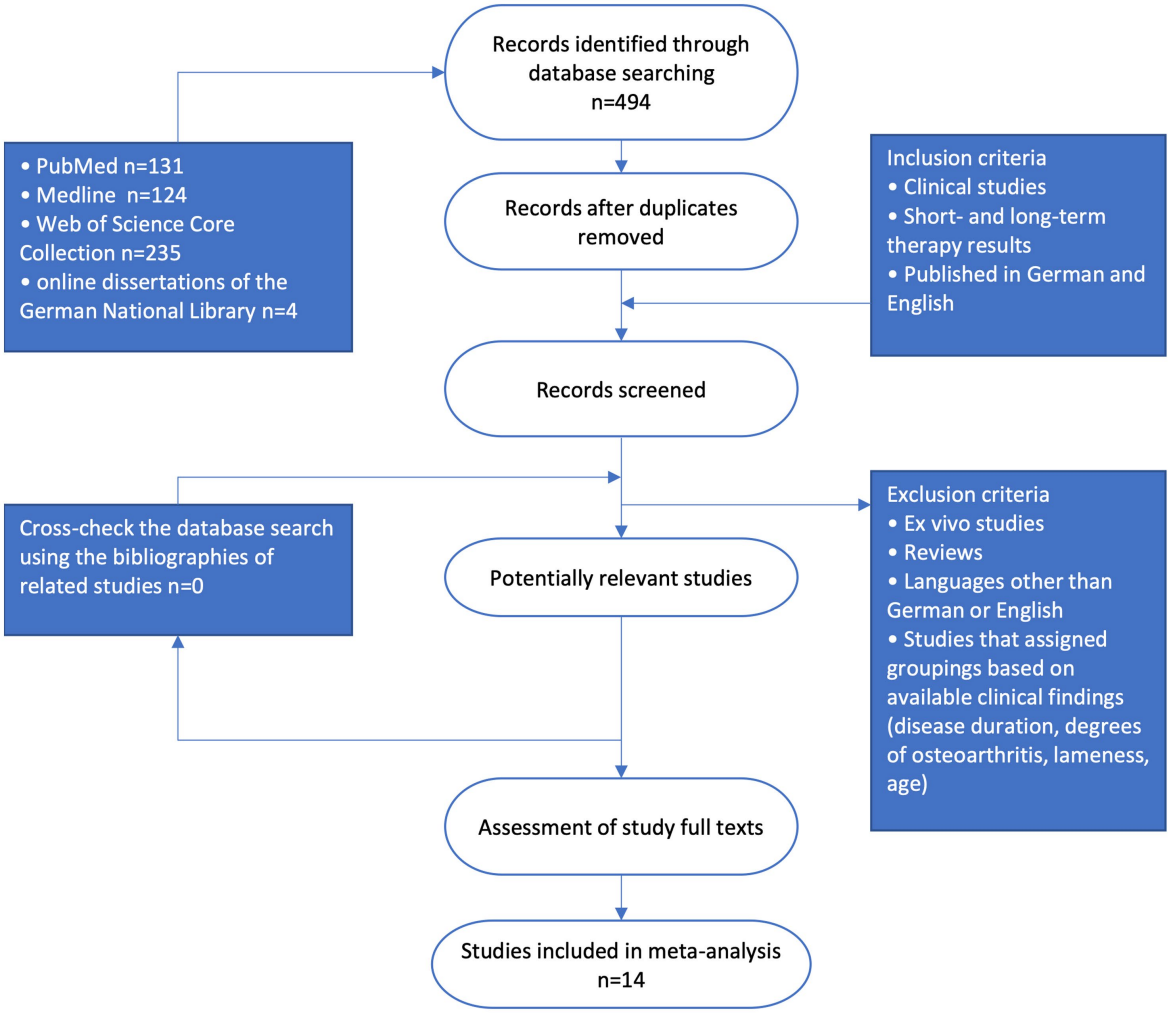
Flow diagram of the literature selection process (PRISMA, participants, intervention, comparison and outcome).

Identification of the relevant literature for this review was performed according to the PRISMA reporting guidelines shown by a flow chart (Figure 1) (6). Randomized, quasi-randomized, and studies that classified their patients based on owner preference or surgeon preference were included. Studies that chose a patients type of intervention based on clinical condition, degrees of osteoarthritis, anamnestic data were excluded to reduce influence of selection bias. Also excluded were reviews, *in vitro* studies, studies in languages other than English and German, studies without available full texts, or without published mid- or long-term results. Anamnestic, diagnostic, clinical, therapeutical and study design data mentioned in these studies pre-intervention, post intervention and at different follow-up times were extracted and recorded in a Microsoft Excel spreadsheet.

### 2.3. Evidence classification

The evidence of individual studies was evaluated according to the Cochrane Musculoskeletal Group Guidelines (7).

### 2.4. Meta-analysis and significance level

Meta-analysis was performed using the integrated development environment and graphical user interface “RStudio” (RRID:SCR_000432) for the R statistical programming language developed by Posit PBC, Boston, United States. *p*-values less than 0.05 were judged to be statistically significant. In all meta-analyses that included outcomes from both minimally invasive and open surgery, subgroup-analyses of arthroscopy and arthrotomy were utilized. This was done to ensure that results from both types of surgical procedures can be viewed separately, avoiding potential distortion in the findings.

### 2.5. Data extraction

The authors of this systematic review ranked multiple intervention results based on their accuracy and lack of bias. For each case, the authors used computerized gait analysis being the top-ranked measure, followed by examination by veterinary staff (veterinarian) and patient owner questionnaire in descending order. Positive surgical results were those in which there was an improvement in the highest relevant criterion (descending order: lameness, painfulness, quality of life, owner satisfaction) compared with the preoperative findings and there were no or only minor complications intra- and post-operatively as defined in each original study. When lameness studies were performed at multiple gaits, the results at trot were used for present meta-analyses.

### 2.6. Dealing with differently specified symmetry indices

Symmetry indices are values—usually given as a percentage or ratio—that describe the symmetry of the subject’s gait pattern. Parameters such as stance phase duration, vertical impulse, and peak vertical force can be determined by computer-assisted gait analysis. Some authors used the affected leg and the contralateral leg of the forelimb to calculate the indices—other authors used the affected leg and either the contra- or ipsilateral leg of the hindlimb. Impulse values were used when available, if not other metrics like maximum force or stance duration were also included. The following symmetry or asymmetry indices (SI) were found in the literature and could be converted into each other and thus compared using the following equations:

Equation 1: Calculation of the symmetry index ratio

(1) $$SI\left(\mathrm{Ratio}\right)=\frac{\mathrm{Xi}}{Xh}$$

*Xi* = ground reaction force of the impaired leg.

*Xh* = ground reaction force of a healthy leg.

Source: Theyse et al. (8).

Equation 2: Calculation of symmetry index 1

(2) $$SI\left(1\right)=\frac{Xh-\mathrm{Xi}}{1/2\left(Xh+\mathrm{Xi}\right)}\times 100$$

Source: Fanchon and Grandjean (9).

Equation 3: Calculation of symmetry index 2

(3) $$SI\left(2\right)=\frac{\bar{\mathrm{Xi}}-\bar{Xh}}{1/2\left(\bar{\mathrm{Xi}}+\bar{Xh}\right)}\times 100$$

$\bar{\mathrm{Xi}}$ = Average ground reaction force of the impaired leg over several steps or trials.

$\bar{Xh}$ = Average ground reaction force of a healthy leg over several steps or trials.

Source: Galindo-Zamora et al. (10) and Volstad et al. (11).

Equation 4: Calculation of symmetry index 3

(4) $$SI\left(3\right)=\frac{\bar{\mathrm{Xi}}-\bar{Xh}}{\bar{\mathrm{Xi}}+\bar{Xh}}\times 100$$

Source: Bockstahler et al. (12).

SI(1) and SI(2) were treated equally and included in the same analysis because previous studies did not find a significant difference between SI(1) and SI(2) (11).

Where neither SI(1) nor SI(2) were available, the authors of this study derived Equation 5 to convert SI(ratio) to SI(1) and Equation 6 to convert SI(3) to SI(1):

Equation 5: Calculation of the symmetry index 1 given symmetry index ratio

(5) $$SI\left(1\right)=\frac{SI\left(\mathrm{Ratio}\right)-1}{\frac{1}{2\left(SI\left(\mathrm{Ratio}\right)+1\right)}}\times 100$$

Equation 6: Calculation of symmetry index 1 given symmetry index 3

(6) $$SI\left(1\right)=2\times SI\left(3\right)$$

Improvement in forelimb loading symmetry was defined as any SI(1) or SI(2) result that showed at least a 5% improvement in symmetry post-surgery compared to pre-surgery. Improvements of less than 5% were also considered if the dog was sound post-surgery, i.e., there was no traceable asymmetry post-surgery (total asymmetry of less than 5% between the affected and healthy leg).

For studies where standard deviation of δSI was not available, the following section used an estimate based on Higgins et al. (13). Using the equation provided (Equation 7), the correlation coefficient is calculated based on the study with the largest sample size (n), that also provides complete data on standard deviations, averages of symmetry indices, and their differences at pre-surgery, long-term post-surgery, and subsequent follow-ups.

Equation 7: Calculation of the correlation coefficient

(7) $$\mathrm{Corr}=\frac{SD_{\mathrm{baseline}}^{2}+SD_{\mathrm{final}}^{2}-SD_{\mathrm{change}}^{2}}{2*SD_{\mathrm{baseline}}^{2}*SD_{\mathrm{final}}^{2}}$$

Source: Higgins et al. (13).

If the correlation coefficient was available, the standard deviation of δSI can be estimated using Equation 8.

Equation 8: Calculation of the change from the base value standard deviation using the correlation coefficient

(8) $$SD_{\mathrm{change}}=\sqrt{SD_{\mathrm{baseline}}^{2}+SD_{\mathrm{final}}^{2}-\left(2*\mathrm{Corr}*SD_{\mathrm{baseline}}\right)}$$

## 3. Results

### 3.1. Database search

A total of 494 papers were found in the databases mentioned above during a search conducted in 2022. After removal of duplicates, 266 papers remained (Table 1). The review of the bibliographies of relevant publications on the topic did not yield any additional publications that had not already been captured by the database search. This result was interpreted as an indicator of careful and complete keyword coverage.

**Table 1:** Number of results of the search by database.

Database	Number of works
Pubmed	131
Web of Science	235
Medline	124
Catalog of the German National Library	4
**Number of publications**	**494**
**Publications after cleaning up duplicates**	**266**

“Number of publications” are the total number acquired by the database search. “Publications after cleaning up duplicates” is the total number after duplicates have been removed.

### 3.2. Identification of relevant literature

After reviewing the abstracts, 103 clinical studies remained that addressed the therapeutic outcome of treatment of elbow dysplasia in dogs. Studies that were manually excluded were those that were not in German or English, *in vitro* studies, those where the full texts were not available, studies that classified the type of therapy based on degrees of osteoarthritis or lameness or did not collect long-term therapy results and studies that included pathology outside the medial elbow compartment. Some studies clearly listed dogs with different pathology and were included when dogs affected by other concomitant disease could be identified and excluded. The selection scheme is shown in Figure 1. Ultimately, 14 papers remained that could be processed into a total of three meta-analyses.

### 3.3. Evidence classification

Out of all the studies evaluated using the Cochrane Musculoskeletal Group Guidelines recommended grading system, only three were deemed to be of silver level evidence (14–16). The remaining fell into the bronze category (8, 10, 17–25), which is considered the lowest level of evidence. None of the studies included in this paper met the criteria for gold or platinum level evidence.

#### 3.3.1. Symmetry index conversion

Results of the conversation of SI (ratio) values given in the work of Theyse et al. (8) to SI (1) values are shown in Table 2.

**Table 2:** Conversion of the SI (ratio) values contained in Theyse et al.

	Ratio SI Iz		SI(1) Iz		Change in SI (1)
Patient	0. day	6. month	0. day	6. month	0. day – 6. month
1	0.96	0.81	−4.28	−20.32	−16.04
2	0.63	0.94	−39.57	−6.42	33.16
3	0.92	0.98	−8.56	−2.14	6.42
4	0.65	0.88	−37.43	−12.83	24.60
5	0.97	0.99	−3.21	−1.07	2.14
6	0.96	0.98	−4.28	−2.14	2.14
7	0.98	0.98	−2.14	−2.14	0.00
Mean	0.87	0.94	−14.21	−6.72	7.49
SD	0.16	0.07	16.73	7.27	16.43

Iz, maximum vertical impulse of a limb; SD, standard deviation; SI, symmetry index.

The correlation coefficient was calculated using Equation 7 based on the study conducted by Theyse et al. (8), yielding a value of 0.00212. This value was utilized in to estimate the standard deviation of the δSI. Consequently, Theyse et al. (8), Galindo-Zamora et al. (10), and Barthelemy et al. (17) obtained standard deviation values of 17.06, 17.64, and 22.40, respectively.

### 3.4. Meta-analysis

The studies included in the meta-analyses are listed in Supplementary Table S1.

#### 3.4.1. Surgical studies without a conservatively treated comparison group

##### 3.4.1.1. Surgical results determined by clinical examination and owner questionnaires

Ten of eleven studies that analyzed outcomes after surgical treatment of MCD without a control group concluded, with a significance of *p* < 0.05, that surgery significantly improved the dog’s quality of life or lameness (10, 18–25). Only within one study (8), the improvements were not significant. None of the studies found a net negative effect of surgery.

**Figure 2:**
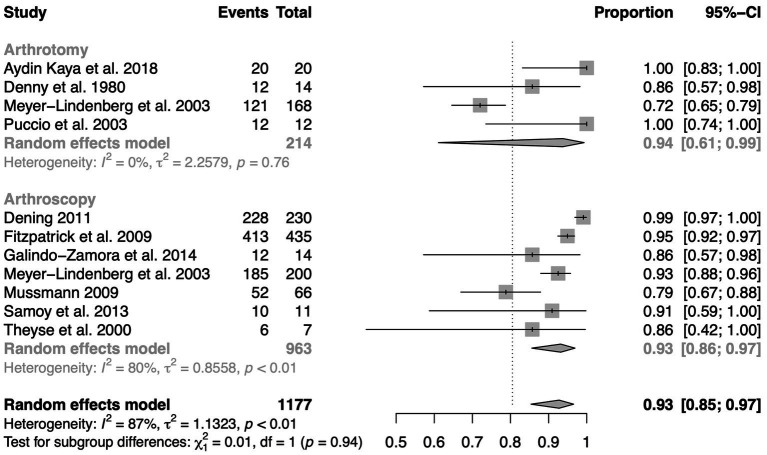
Forest plot diagram of success rates (satisfactory surgical outcome or better) grouped by arthroscopic and arthrotomic intervention of MCD.

The meta-analysis of success rates (success is considered to be at least a satisfactory surgical result) concludes with high significance (*p* < 0.01) that within the totality of all studies, surgical treatment leads to high owner satisfaction and subjectively improved lameness of the dog (Figure 2).

##### 3.4.1.2. Symmetry according to computer-aided analysis of ground reaction forces

**Figure 3:**
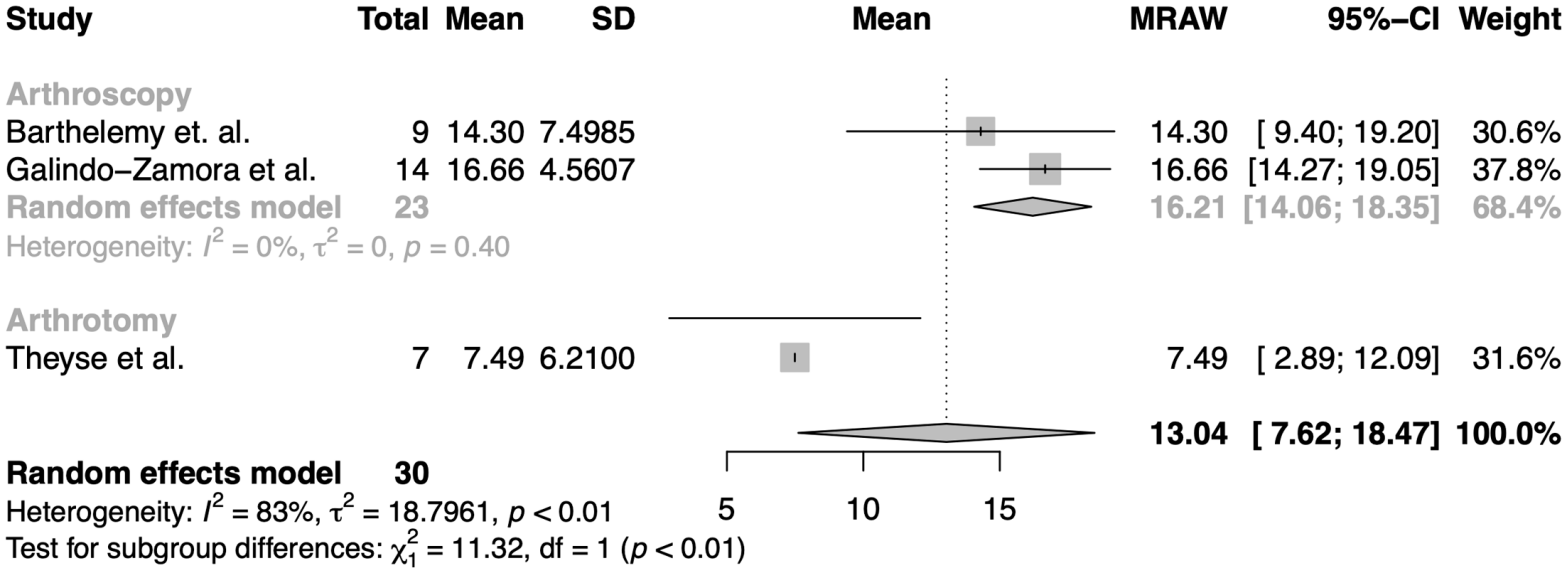
Meta-analysis of differences in mean improvements in the symmetry index compared to the pre-surgery state determined by computer-assisted gait analysis after arthroscopy (arthrotomy in the case of Theyse et al.) in the presence of MCD.

Gait analysis compared the studies of Theyse et al. (8), Galindo-Zamora et al. (10), Barthelemy et al. (17). According to Theyse et al., six out of seven dogs were sound or had improved lameness status at 180 days post-surgery. Nevertheless, the results in improved symmetry were not statistically significant. And similarly, the results of Barthelemy et al. were also not statistically significant. In comparison, Galindo-Zamora et al. found statistically significant improvement in the symmetry index or freedom from any lameness postoperatively in 12 of 14 dogs (*p* < 0.01). Also statistically significant was the improvement of the asymmetry index in the meta-analysis performed including all three studies (*p* < 0.01) (Figure 3).

#### 3.4.2. Surgical studies with conservative comparison group

**Figure 4:**
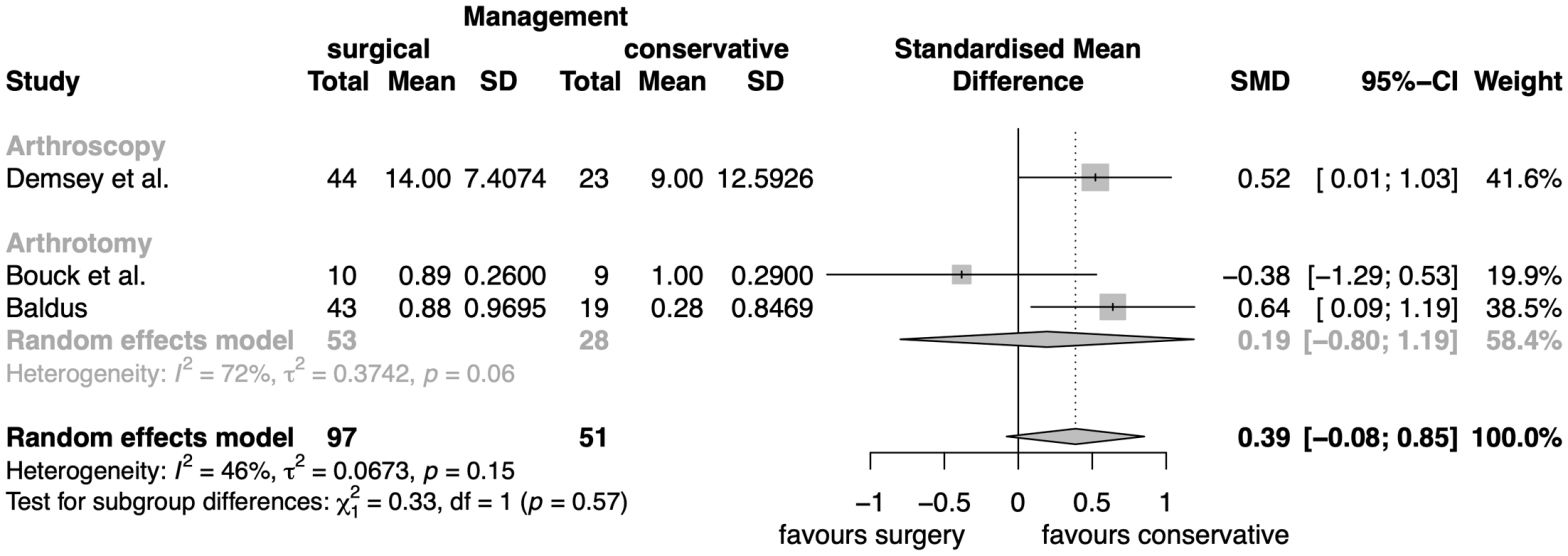
Meta-analysis of standardized mean difference of lameness scores between conservative and surgical management of MCD.

This meta-analysis included studies that compared conservative and surgical intervention (Figure 4). A total of 97 elbows were treated surgically by arthroscopy or arthrotomy with 51 being treated medically. With a *p*-value of 0.15, it failed to find a significant difference between conservative and surgical therapy, specifically arthroscopy or arthrotomy in the presence of medial compartment disease.

## 4. Discussion

This systematic review evaluated 14 studies on outcomes of therapy for canine medial elbow compartment disorders. Thus, this study is more extensive than an earlier systematic review from the year 2008 with a total of four studies (26).

The first meta-analysis of the success rate (Figure 2) attests surgery a high percentage of satisfactory to very good results. The study design of the underlying publications is however susceptible to observer bias and placebo effect. Owners and veterinarians assessing the lameness and pain condition may overestimate the effect of the surgery compared to the actual improvement due to their expectations.

The second meta-analysis of the mean difference between pre-surgery and post-surgery symmetry indices is not susceptible to observer bias and placebo effect, as these values were computerized. Accordingly, there is clear evidence that surgical intervention produces an improvement in lameness (*p* < 0.01). Nevertheless, this meta-analysis does not explore the relative efficacy of surgery in comparison to non-invasive conservative therapy approaches.

This is addressed utilizing standardized mean difference by the third meta-analysis which probes into the disparities in lameness levels stemming from conservative and surgical therapies. Its conclusion drawn suggests that the existing evidence is inadequate to affirm a considerable disparity between non-invasive medical and surgical management. But there is also a possibility of systematic errors occurring in this meta-analysis: The way in which subjects are divided into the different groups is problematic. Even though studies that classified their subjects based on degrees of lameness and arthrosis were excluded from the outset, the included studies partially used no selection mechanisms to assign patients to groups that were based on true randomness. For instance, allocating the patient to its group by presentation time with the first half of the dogs being assigned to group I and all subsequent patient to group II carries a high risk for selection bias. It does not represent a valid randomization mechanism, because although the balance may be guaranteed, the next allocation is easily recognizable. This form of randomization, called quasi-randomization, must be distinguished in quality from truly random procedures. And secondly, the number of studies (*n* = 3) and the total number of elbows (*n* = 148) were limited. The low sample size is especially problematic when looking at the results of subgroup analyses where the number of dogs was reduced even further. Looking at the combined outcomes of both surgical entities is not optimal either because it can lead to substantial distortion of the overall surgical results.

Also, all three meta-analyses evaluated show a tendency to systematic errors since studies dealing with surgery outcomes are usually not double-blinded for ethical reasons. This is in line with the assessment of evidence using the Cochrane Musculoskeletal Group Guidelines’ evidence classification system. All the studies that formed part of the three meta-analyses had a quality of evidence of bronze or silver level only, considerably lessening their interpretive power.

Meta-analyses can only ever be as good as the primary studies available to them (27). Nevertheless, this work highlights that surgical intervention of medial compartment disease performs favorably over the pre-surgery condition in non-controlled pre-surgery-post-surgery cross-over trials that determined owner satisfaction, as well as lameness levels or performed objective gait analyses.

However, when directly comparing conservative and surgical therapy in studies with groups of different treatment approaches, the situation is complicated. A general superiority or inferiority of surgical compared to conservative management was not proven with the help of this meta-analysis due to lack of significance. This is in agreement with the assessment of other authors who then followed up saying that the decision for or against surgical therapy remains a question of the severity of the disease, the progression of the osteoarthritis, the age of the patient and ultimately also the preference of the surgeon (14, 28). Unassailable evidence of these proposals, however, remains to be found. The authors of this article do not want to rule out that superiority of surgical therapy may in the future be clearly proven. This could be in certain breeds, age groups and specific radiologic or arthroscopic findings even though prior research concluded that radiographs as well as computed tomography are limited in finding erosions of the medial elbow compartment (29). Further studies with higher numbers of subjects, detailed anamnestic documentation on race, age, body condition score, duration of disease, degrees of osteoarthritis and existing concomitant disease will be required. It is expected that reliable recommendations can then be made to assist clinician and patient owner in their decision finding process. Given the results presented and the limited availability of high-quality studies, the authors believe it is plausible that for many affected dogs, conservative therapy could be on par with surgery. And as long as there is absence of strong evidence supporting the superiority of surgical treatment, conservative therapy might more often be the sensible approach for both ethical and financial reasons.

## Data availability statement

The original contributions presented in the study are included in the article/Supplementary material, further inquiries can be directed to the corresponding author.

## Author contributions

HK contributed to the conception and study design, development of methodology, database search, identification of relevant literature, evidence classification, as well as draft, revisions, approval, and submission of the article. YZ and HK conducted the statistical analysis (meta-analysis). AM-L contributed to the supervision, revision of the article for intellectual content, and approval of the final article. All authors contributed to the article and approved the submitted version.

## References

[1] Meyer-Lindenberg A Fehr M Nolte I. Co-existence of ununited anconeal process and fragmented medial coronoid process of the ulna in the dog. J Small Anim Pract. (2006) 47:61–5. doi: 10.1111/j.1748-5827.2006.00051.x, PMID: 16438692

[2] Group IEW. About Iewg. (2023). Available at: http://www.vet-iewg.org/about/.

[3] Van Ryssen B van Bree H. Arthroscopic findings in 100 dogs with elbow lameness. Vet Rec. (1997) 140:360–2. doi: 10.1136/vr.140.14.360, PMID: 9133719

[4] Vermote KAG Bergenhuyzen ALR Gielen I van Bree H Duchateau L Van Ryssen B. Elbow lameness in dogs of six years and older: arthroscopic and imaging findings of medial coronoid disease in 51 dogs. Vet Comp Orthop Traumatol. (2010) 23:43–50. doi: 10.3415/VCOT-09-03-0032, PMID: 19997673

[5] Fitzpatrick N Smith TJ Evans RB Yeadon R. Radiographic and arthroscopic findings in the elbow joints of 263 dogs with medial coronoid disease. Vet Surg. (2009) 38:213–23. doi: 10.1111/j.1532-950X.2008.00489.x 19236680

[6] Moher D Shamseer L Clarke M Ghersi D Liberati A Petticrew M . Preferred reporting items for systematic review and meta-analysis protocols (PRISMA-P) 2015 statement. Syst Rev. (2015) 4:1. doi: 10.1186/2046-4053-4-1, PMID: 25554246 PMC4320440

[7] Maxwell L Santesso N Tugwell PS Wells GA Judd M Buchbinder R. Method guidelines for Cochrane musculoskeletal group systematic reviews. J Rheumatol. (2006) 33:2304–11. PMID: 17086611

[8] Theyse LFH Hazewinkel HAW van den Brom WE. Force plate analyses before and after surgical treatment of unilateral fragmented coronoid process. Vet Comp Orthop Traumatol. (2000) 13:135–40. doi: 10.1055/s-0038-1632648

[9] Fanchon L Grandjean D. Accuracy of asymmetry indices of ground reaction forces for diagnosis of hind limb lameness in dogs. Am J Vet Res. (2007) 68:1089–94. doi: 10.2460/ajvr.68.10.1089 17916016

[10] Galindo-Zamora V Dziallas P Wolf DC Kramer S Abdelhadi J Lucas K . Evaluation of thoracic limb loads, elbow movement, and morphology in dogs before and after arthroscopic management of unilateral medial coronoid process disease. Vet Surg. (2014) 43:819–28. doi: 10.1111/j.1532-950X.2014.12250.x, PMID: 25073482

[11] Volstad NJ Sandberg G Robb S Budsberg SC. The evaluation of limb symmetry indices using ground reaction forces collected with one or two force plates in healthy dogs. Vet Comp Orthop Traumatol. (2017) 30:54–8. doi: 10.3415/vcot-16-04-0054 27849103

[12] Bockstahler BA Vobornik A Müller M Peham C. Compensatory load redistribution in naturally occurring osteoarthritis of the elbow joint and induced weight-bearing lameness of the forelimbs compared with clinically sound dogs. Vet J. (2009) 180:202–12. doi: 10.1016/j.tvjl.2007.12.025, PMID: 18406183

[13] Higgins JPTTJ Chandler J Cumpston M Li T Page MJ Welch VA. Cochrane handbook for systematic reviews of interventions version 5.2 The Cochrane Collaboration (2017).

[14] Bouck GR Miller CW Taves CL. A comparison of surgical and medical treatment of fragmented coronoid process and osteochondritis dissecans of the canine elbow. Vet Comp Orthop Traumatol. (1995) 8:177–83. doi: 10.1055/s-0038-1632452

[15] Dempsey LM Maddox TW Comerford EJ Pettitt RA Tomlinson AW. A comparison of owner-assessed long-term outcome of arthroscopic intervention versus conservative management of dogs with medial coronoid process disease. Vet Comp Orthop Traumatol. (2019) 32:001–9. doi: 10.1055/s-0038-1676293, PMID: 30646406

[16] Baldus IMV. Langzeitergebnisse Nach Arthroskopischer Therapie Der Koronoiderkrankung Im Vergleich Zu Den Ellbogengelenken Konservativ Behandelter Hunde [Dissertation]. Gießen: Justus-Liebig-Universität (2013).

[17] Barthelemy NP Griffon DJ Ragetly GR Carrera I Schaeffer DJ. Short- and long-term outcomes after arthroscopic treatment of young large breed dogs with medial compartment disease of the elbow. Vet Surg. (2014) 43:935–43. doi: 10.1111/j.1532-950X.2014.12255.x, PMID: 25088613

[18] Samoy YC de Bakker E Van Vynckt D Coppieters E van Bree H Van Ryssen B. Arthroscopic treatment of fragmented coronoid process with severe elbow incongruity. Long-term follow-up in eight Bernese Mountain dogs. Vet Comp Orthop Traumatol. (2013) 26:27–33. doi: 10.3415/vcot-11-06-0087, PMID: 23154671

[19] Fitzpatrick N Smith TJ Evans RB O'Riordan J Yeadon R. Subtotal coronoid ostectomy for treatment of medial coronoid disease in 263 dogs. Vet Surg. (2009) 38:233–45. doi: 10.1111/j.1532-950X.2008.00491.x, PMID: 19236682

[20] Puccio M Marino DJ Stefanacci JD McKenna B. Clinical evaluation and long-term follow-up of dogs having coronoidectomy for elbow incongruity. J Am Anim Hosp Assoc. (2003) 39:473–8. doi: 10.5326/0390473, PMID: 14518656

[21] Dening R. Untersuchungen Zur Therapie Des Fragmentierten Processus Coronoideus Medialis Der Ulna Des Hundes: Einfluss Bestehender Intraartikulärer Veränderungen Auf Das Therapieergebnis/Ricarda Dening [Dissertation]. Hannover: Tierärztliche Hochschule Hannover (2011).

[22] Mussmann K. Ellbogengelenkdysplasie Des Hundes: Studie Zur Bildgebenden Diagnostik Und Postoperativen Erfolgskontrolle Mittels Computerisierter Ganganalyse [Dissertation]. München: LMU (2009).

[23] Meyer-Lindenberg A Langhann A Fehr M Nolte I. Arthrotomy versus arthroscopy in the treatment of the fragmented medial coronoid process of the ulna (FCP) in 421 dogs. Vet Comp Orthop Traumatol. (2003) 16:204–10. doi: 10.1055/s-0038-1632780

[24] Denny HR Gibbs C. The surgical-treatment of osteochondritis dissecans and ununited coronoid process in the canine elbow joint. J Small Anim Pract. (1980) 21:323–31. doi: 10.1111/j.1748-5827.1980.tb01254.x 7431879

[25] Aydin Kaya D Altunatmaz K. The clinical and radiological evaluation of medial coronoid disease in dogs: 20 cases. Kafkas Univ Vet Fak Derg. (2018) 24:709–16. doi: 10.9775/kvfd.2018.19772

[26] Evans RB Gordon-Evans WJ Conzemius MG. Comparison of three methods for the management of fragmented medial coronoid process in the dog. A systematic review and meta-analysis. Vet Comp Orthop Traumatol. (2008) 21:106–9. doi: 10.3415/VCOT-07-04-0031, PMID: 18545711

[27] Sharpe D. Of apples and oranges, file drawers and garbage: why validity issues in meta-analysis will not go away. Clin Psychol Rev. (1997) 17:881–901. doi: 10.1016/s0272-7358(97)00056-1 9439872

[28] Burton NJ Owen MR Kirk LS Toscano MJ Colborne GR. Conservative versus arthroscopic management for medial coronoid process disease in dogs: a prospective gait evaluation. Vet Surg. (2011) 40:972–80. doi: 10.1111/j.1532-950X.2011.00900.x, PMID: 22091562

[29] Moores AP Benigni L Lamb CR. Computed tomography versus arthroscopy for detection of canine elbow dysplasia lesions. Vet Surg. (2008) 37:390–8. doi: 10.1111/j.1532-950X.2008.00393.x, PMID: 18564264

