# Supplementary material for: Therapeutic success in fragmented coronoid process disease and other canine medial elbow compartment pathology: a systematic review with meta-analyses
Source: Front Vet Sci. 2023 Nov 9;10:1228497. doi: 10.3389/fvets.2023.1228497 (PMC10666176; doi:10.3389/fvets.2023.1228497)
Supplement: Supplementary file 1 [file Data_Sheet_1.pdf]

Supplementary Material

Therapeutic Success in Fragmented Coronoid Process Disease and Other Canine Medial Elbow Compartment Pathology: A Systematic Review with Meta-Analyses

Hubertus Kähn[1], Yury Zablotski[1], Andrea Meyer-Lindenberg[1]

*** Correspondence:** Hubertus Kähn: h.kaehn@lmu.de

Supplementary data 1: Search phrase Pubmed

("Dogs"[Mesh] OR dog[tw] OR dogs[tw] OR canine*[tw]) AND ("fragmented coronoid process*"[tw] OR "fragmented medial coronoid process*"[tw] OR "coronoid disease*"[tw] OR "medial coronoid*"[tw] OR "coronoid process*"[tw] OR "medial compartment disease*"[tw] OR "medial compartment syndrome*"[tw] OR "jump-down syndrome*"[tw] OR "fractured coronoid*"[tw] OR "coronoid fracture*"[tw] OR "ununited coronoid*"[tw]) AND ("ulna osteotom*"[tw] OR "ulnar osteotom*"[tw] OR "coronoid ostectom*"[tw] OR "coronoidectom*"[tw] OR "arthroscop*"[tw] OR "elbow replacement*"[tw] OR "joint replacement*"[tw] OR "arthroplast*"[tw] OR "elbow resurfacing"[tw] OR "humeral osteotom*"[tw] OR "humerus osteotom*"[tw] OR "treatment*"[tw] OR "surger*"[tw] OR "removal*"[tw] OR "biceps tendon release" OR "biceps ulnar release*"[tw] OR "ulnar release procedure*"[tw] OR "therap*"[tw])

Supplementary data 2: Search phrase Web of Science

((TS=((Dog* OR canine*)])) AND TS=(("fragment* coronoid *process*" OR "fragment* media* coronoid* process*" OR "coronoid disease*" OR "medial coronoid*" OR "coronoid process*" OR "media* compartment* disease*" OR "media* compartment* syndrome*" OR "jump-down syndrome*" OR "fracture* coronoid*" OR "coronoid* fracture*" OR "ununit* coronoid*"))) AND TS=(("ulna osteotom*" OR "ulnar osteotom*" OR "coronoid ostectom*" OR "coronoidectom*" OR "arthroscop*" OR "elbow replacement*" OR "joint replacement*" OR "arthroplast*" OR "elbow resurfacing" OR "humeral osteotom*" OR "humerus osteotom*" OR "treatment*" OR "surger*" OR "removal*" OR "biceps tendon release" OR "biceps ulnar release*" OR "ulnar release procedure*" OR "therap*"))

Supplementary data 3: Search phrase MEDLINE

(exp Dogs/ or dog.mp. or dogs.mp. or canine*.mp.) and ("fragment* coronoid* process*".mp. or "fragment* media* coronoid* process*".mp. or "coronoid* disease*".mp. or "media* coronoid*".mp. or "coronoid* process*".mp. or "media* compartment* disease*".mp. or "media* compartment* syndrome*".mp. or jump-down syndrome*".mp. or fracture* coronoid*".mp. or "coronoid* fracture*".mp. or "ununit* coronoid*".mp.) and ("ulna* osteotom*".mp. or "coronoid* ostectom*".mp. "coronoidectom*".mp. or "arthroscop*".mp. or "elbow* replacement*".mp. or "joint* replacement*".mp. or "arthroplast*".mp. or "elbow* resurfacing*".mp. or "humer* osteotom*".mp. or "treatment*".mp. or "surger*".mp. or "removal*".mp. or "bicep* tendon* release*".mp. or "bicep* ulna* release*".mp. or "ulna* release* procedure*".mp. "therap*".mp.)

Supplementary Table 1

Study				Patients					Included diseases							Comparison group	Interventions				Follow-up										Meta-analysis			Diagnosis							Correct identification of elbow pathology (FCP, MCD, etc.).
Name	Author	Year	Level of evidence (CMSG)	Age from (months)	Age until (months)	Age Ø (months)	Number of dogs	Elbow	MCD	FCP	OCD	Elbow Incongruincy	UAP	OA IEWG SCORE	Other diseases	if yes type of division:	Arthroscopy	Arthrotomy	Ulnar Osteotomy	Conservative	Computerized gait analysis	Clinical muscle	Goniometry	Clinical painfulness	Clinical lameness	Owner questionnaire	Radiological	First (months)	Last (months)	Ø (months)	Symmetry indices	Clinical US + owner	Conservative vs Surgical	Radiological	CT	MRI	Clinical	Arthroscopic	Arthrotomic	Szintigraphy	
Short- and long-term outcomes after arthroscopic treatment of young large breed dogs with medial compartment disease of the elbow	Barthelemy, Nicolas P., Griffon, Dominique J., Ragetly, Guillaume R., Carrera, Ines, Schaeffer, David J.	2014	bronze	0	36	13	15	23	Yes	Yes	n.a.	Yes	No	n.a.	No	No	Yes	No	Yes	No	Yes	Yes	Yes	No	No	No	Yes	1,5	>23		Yes	No	No	Yes	Yes	No	Yes	Yes	No	No	No
Evaluation of thoracic limb loads, elbow movement, and morphology in dogs before and after arthroscopic management of unilateral medial coronoid process disease	Galindo-Zamora, Vladimir, Dziallas, Peter, Wolf, Davina C., Kramer, Sabine, Abdelhadi, Jalal, Lucas, Karin, Nolte, Ingo, Wefstaedt, Patrick	2014	bronze	10	120	61	14	14	Yes	Yes	No	No	No	n.a.	No	No	Yes	No	No	No	Yes	No	No	No	No	No	No	2	6,5		Yes	Yes	No	Yes	Yes	No	Yes	Yes	No	No	No
Force plate analyses before and after surgical treatment of unilateral fragmented coronoid process.	Theyse, L. F. H., Hazewinkel, H. A. W.,van den Brom, W. E.	2000	bronze	7	66		7	7	Yes	Yes	No	No	No	0-3	No	No	No	Yes	No	No	Yes	No	No	No	No	No	No	1,5	6*		Yes	No	No	Yes	No	No	Yes	No	Yes	No	No
Arthroscopic treatment of fragmented coronoid process with severe elbow incongruity. Long-term follow-up in eight Bernese Mountain Dogs	Samoy, Y. C., de Bakker, E., Van, Vynckt, D., Coppieters, E., van Bree, H., Van Ryssen, B..	2013	bronze	0	24	n.a.	8	11	Yes	Yes	n.a.	Yes	No	2-3	No	No	Yes	No	No	No	No	Yes	Yes	Yes	Yes	No		49	90	67	No	Yes	No	Yes	Yes	No	Yes	Yes	No	No	Yes, cartilage erosion has been described
Subtotal coronoid ostectomy for treatment of medial coronoid disease in 263 dogs	Fitzpatrick, Noel, Smith, Thomas J., Evans, Richard B., O'Riordan, Jerry, Yeadon, Russell	2009	bronze	4	135	32	246	435	**Yes**	Yes	No	No	No	n.a.	No	No	Yes*	No	No	No	No	No	Yes	Yes	Yes	Yes	Yes			19	No	Yes	No	Yes	No	No	No	Yes	No	No	Yes, cartilage erosion has been described
Clinical evaluation and long-term follow-up of dogs having coronoidectomy for elbow incongruity	Puccio, Margaret, Marino, Dominic J., Stefanacci, Joseph D., McKenna, Brian	2003	bronze	9	48	25	17	18	No	No	No	**Yes**	No	1-3	No	No	No	Yes	No	No	No	No	No	No	Yes	No		1	38		No	Yes	No	Yes	No	No	Yes	No	Yes	No	Yes, cartilage erosion has been described
Untersuchungen zur Therapie des fragmentierten Processus coronoideus medialis der Ulna des Hundes: Einfluss bestehender intraartikulärer Veränderungen auf das Therapieergebnis / Ricarda Dening	Dening, Ricarda	2011	bronze	4,8	132	25	200	230	Yes	Yes	Yes	No	No	0-3	No	No	Yes	No	No	No	No	Yes	No	Yes	Yes	Yes	Yes	6	24		No	Yes	No	Yes	No	No	Yes	Yes	No	No	Mention of chondromalacia and cartilaginous ulcers
Ellbogengelenkdysplasie des Hundes: Studie zur bildgebenden Diagnostik und postoperativen Erfolgskontrolle mittels computerisierter Ganganalyse	Mussmann, Karin	2009	bronze	5,3	102	39	52	61	Yes	Yes	Yes	Yes	Yes	n.a.	No	No	No	Yes	Yes	No	Yes	No	No	No	Yes	No	Yes	6	80	32	No	Yes	No	Yes	Yes	No	Yes	No	Yes	No	MCD not mentioned
A Comparison of Surgical and Medical Treatment of Fragmented Coronoid Process and Osteochondritis Dissecans of the Canine Elbow	Bouck, G. R., Miller, C. W., Taves, C. L..	1995	silver	7	28	13	19	19	Yes	Yes	Yes	No	No	n.a.	No	Randomized	No	Yes	No	Yes	Yes	No	Yes	Yes	Yes	No	Yes	2	9	n.a.	No	No	Yes	Yes	No	No	Yes	No	Yes	No	No
A Comparison of Owner-Assessed Long-Term Outcome of Arthroscopic Intervention versus Conservative Managemento f Dogs with Medial Coronoid Process Disease.	Dempsey, Lara M., Maddox, Thomas W., Comerford, Eithne J., Pettitt, Rob A., Tomlinson, Andrew W.	2019	silver	5	64		67	67	Yes	Yes	No		No	n.a.	No	Owner or surgeon desire, financial criteria.	Yes	No	No	Yes	No	No	No	Yes	Yes	Yes	Yes	12	n.a.	n.a.	No	No	Yes	Yes	Yes	No	Yes	Yes	No	No	No
Conservative versus arthroscopic management for medial coronoid process disease in dogs: a prospective gait evaluation	Baldus, Inga	2011	silver	6	158	23	62	100	Yes	Yes	Yes	Yes	No	n.a.	No	Owner request	Yes	No	No	Yes	No		Yes	Yes	Yes	Yes	Yes	0,5	66	n.a.	No	No	Yes	Yes	No	No	Yes	Yes	No	No	No
Arthrotomy versus arthroscopy in the treatment of the fragmented medial coronoid process of the ulna (FCP) in 421 dogs.	A. Meyer-Lindenberg, A. Langhann, M. Fehr and I. Nolte	2003	bronze	5	132	23		200	No	Yes	Yes	Yes	No	0-3	No	No	Yes	No	Yes	No	No	No		Yes	Yes	Yes		6	n.a.	n.a.	No	Yes	No	Yes	No	No	Yes	Yes	No	No	MCD not mentioned
Arthrotomy versus arthroscopy in the treatment of the fragmented medial coronoid process of the ulna (FCP) in 421 dogs.	A. Meyer-Lindenberg, A. Langhann, M. Fehr and I. Nolte	2003	bronze	5	132	23		168	No	Yes	Yes	Yes	No	0-3	No	No	No	Yes	Yes	No	No	No		Yes	Yes	Yes		6	n.a.	n.a.	No	Yes	No	Yes	No	No	Yes	No	Yes	No	MCD not mentioned
The surgical treatment of osteochondritis dissecans and ununited coronoid process in the canine elbow joint	Denny, Gibbs	1980	bronze	4	8	6	7	8	No	Yes	Yes	No	No	No	No	No	No	Yes	No	No	No	No		No	Yes	No		3	36	n.a.	No	Yes	No	Yes	No	No	Yes	No	Yes	No	MCD not mentioned
The Clinical and Radiological Evaluation of Medial Coronoid Diase in Dogs: 20 Cases	Aydin Kaya, Altunatmaz	2018	bronze	7	36	11	16	16	No	Yes	No	No	No	No	No	No	No	Yes	No	No	No	No		No	Yes	Yes		1	2	n.a.	No	Yes	No	16/16	2/16	No	Yes	2/16	Yes	No	*MCD not mentioned
